# The Unforeseen Diagnosis: Hyperparathyroidism-Jaw Tumour Syndrome Case Report and Review of the Literature

**DOI:** 10.1155/2021/5551203

**Published:** 2021-05-20

**Authors:** Maxim Barnett, Farhan Ahmed, Radu Mihai, Asha Rattan, Malik Asif Humayun

**Affiliations:** ^1^University of Buckingham Medical School, Hunter St, Buckingham MK18 1EG, UK; ^2^Milton Keynes University Hospital, Standing Way, Eaglestone, Milton Keynes MK6 5LD, UK; ^3^Oxford Radcliffe Hospitals NHS Foundation Trust, Old Road, Headington, Oxford OX3 7LH, UK

## Abstract

Hypercalcaemia and its systemic sequelae are a relatively common finding amongst patients in the field of endocrinology. Primary hyperparathyroidism, a frequent cause of hypercalcaemia, is often seen among middle-aged female patients, typically resulting from an underlying single-gland adenoma. Although patients may present with symptoms (nephrolithiasis, musculoskeletal discomfort, dehydration, or mood disturbance, to name a few), hypercalcaemia is rather frequently identified incidentally. In younger patients, a familial form of primary hyperparathyroidism must be considered, with a positive diagnosis mandating familial screening. Hyperparathyroidism-jaw tumour syndrome is one such autosomal dominant familial disorder, characterised by a mutation in the cell division cycle 73 (CDC73; also known as HRPT-2) tumour suppressor gene. This disorder is characterised by multiple pleiotropic phenomena, including recurrent primary hyperparathyroidism (and the effects of hypercalcaemia), neoplasms (such as uterine, renal, mandibular, and maxillary), and infertility. A patient not conforming to the classic candidacy for primary hyperparathyroidism requires consideration for a familial cause. *Case Description*. We present a rare diagnostic entity—hyperparathyroidism-jaw tumour (HPT-JT) syndrome—in a 36-year-old female with recurrent primary hyperparathyroidism, frequent nephrolithiasis, and infertility for 18 years prior to the diagnosis. We aim to promote awareness amongst medical professionals of this rare, but nonetheless essential differential diagnosis through a case report and review of the literature. *Conclusion*. Medical professionals must avoid diagnostic overshadowing and display a low threshold for genetic testing in younger patients with primary hyperparathyroidism. The importance of proper identification extends beyond the patient to their relatives and offspring.

## 1. Introduction

Hypercalcaemia, defined as a serum calcium level greater than 2.6 mmol/L (10.4 mg/dL) [1], is a frequent finding amongst patients in the field of endocrinology. Whilst most patients are identified incidentally, hypercalcaemia is commonly a manifestation of primary hyperparathyroidism, a condition characterised by excessive parathyroid hormone release. This disorder is common, with an estimated prevalence of 1/1,000, and is typically diagnosed in female adults (average age pooled at 55 years) [2]. In the presence of a young patient with symptoms suggestive of primary hyperparathyroidism, a familial cause must be considered. This paper, which presents a genetically confirmed case of hyperparathyroidism-jaw tumour syndrome, is written to enhance the awareness of this diagnosis and to reaffirm the requirements to screen for a familial cause in adolescent primary hyperparathyroidism.

## 2. Case Presentation

A 36-year-old female of eastern European origin is reviewed in the urology department in 2018 for a follow-up appointment due to a unique history of eight prior lithotripsy procedures (between the years 2004 and 2011) for prior (recurrent) renal stones. A CT-KUB (computed tomography of the kidneys, ureters, and bladder) had been performed, which identified nephrocalcinosis—not nephrolithiasis. The urology department recommended no further intervention and referred her to the metabolic medicine department for biochemical evaluation.

### 2.1. Past Medical History

Further history taking in the metabolic medicine clinic identified a prior diagnosis of primary hyperparathyroidism in 2003 (age 21) for symptomatic, uncontrollable hypercalcaemia and severe osteoporosis. A parathyroidectomy had been performed with the removal of a large ectopic parathyroid adenoma located behind the right clavicle, with subsequent calcium and parathyroid hormone normalisation. Despite the uncharacteristically young age of the patient, as there was no family history of primary hyperparathyroidism nor familial neoplastic conditions, a familial cause was considered “unlikely” and was not investigated further. Postparathyroidectomy, the patient was prescribed 2,000 units daily of vitamin D and was placed under annual follow-up. Her annual follow-up remained uneventful; however, over the past three years (2014–2017), her surgical team had identified mild, nonprogressive hypercalcaemia. The hypercalcaemia remained static, and a recurrence of primary hyperparathyroidism was ruled out with an unremarkable sestamibi scan (failing to identify an adenoma) and subsequent dual-energy X-ray absorptiometry scan (demonstrating normal bone densitometry).

Her only other significant past medical history includes the removal of uterine leiomyomas, and she has (unsuccessfully) attempted to conceive on multiple occasions with her husband, for which she is under the care of the obstetrics and gynaecology department for infertility.

### 2.2. Investigations

Within the metabolic medicine clinic, a comprehensive metabolic panel was requested. Her serum calcium returned elevated (2.65 mmol/L), in addition to an inappropriately increased parathyroid hormone (9.5 pmol/L) and hypophosphataemia (0.7 mmol/L). Although a reduction in renal function was noted (eGFR 57 mL/min), this was a chronic finding and was not considered significant. As her vitamin D levels were replete (99 nmol/L), replacement was not required and further confirmatory tests were ordered, including a urine calcium-to-creatinine ratio, 24-hour urinary calcium, and fractional excretion of calcium, all of which returned elevated, consistent with a recurrence of primary hyperparathyroidism (Table 1).

Despite an absent positive family history of primary hyperparathyroidism, a genetic panel was requested due to her young age and recurrence of the disease. She was referred to Churchill Hospital (Oxford), whereby MLPA (multiplex ligation-dependent probe amplification) analysis was performed, assessing for eight known genetic mutations responsible for familial forms of primary hyperparathyroidism (Table 2).

To our surprise, the analysis returned with a demonstration of a heterozygous deletion of exons 8-9 (CDC73 (HRPT-2) gene), confirming hyperparathyroidism-jaw tumour (HPT-JT) syndrome. The patient was confused as to why there are no other family members afflicted if this is a familial disorder; it was explained the disease may have occurred from a “de novo” mutation or is present in a family member with incomplete penetrance. The patient's family members have been notified and have undergone screening for the mutation.

### 2.3. Treatment and Outcome

**Figure 1:**
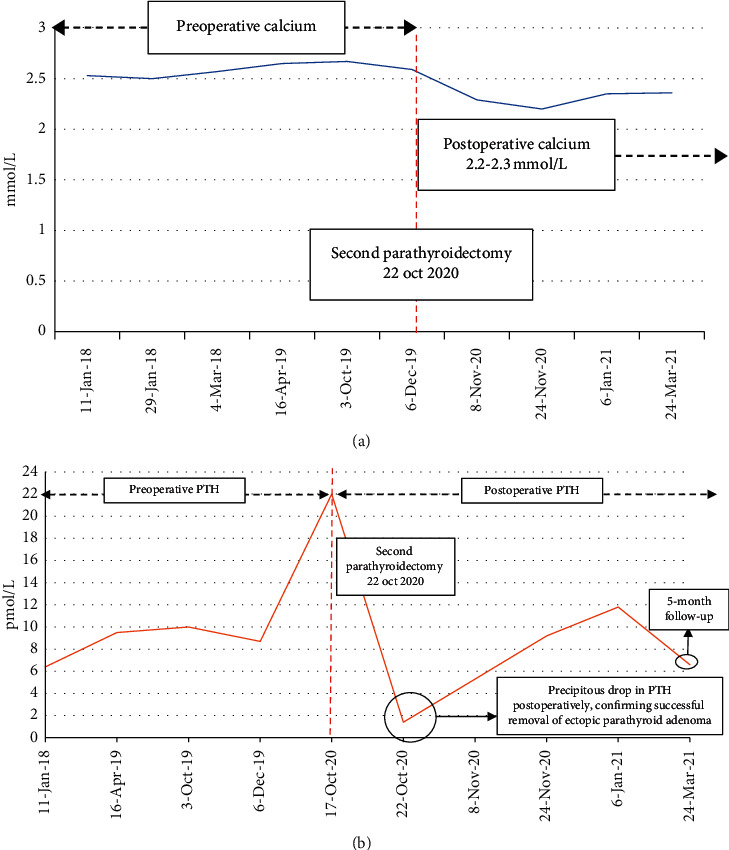
Calcium and parathyroid hormone (PTH) before and after second parathyroidectomy.

A repeat sestamibi-SPECT (single-photon emission computed tomography) scan was performed, demonstrating a 5 mm enhancing nodule deep to the right thyroid lobe. During the second parathyroidectomy procedure (October 2020) however, the surgical team identified a mass on the right side of the thymus (and not deep to the thyroid lobe as the scan had suggested), which was fully excised and sent for histopathological analysis; the mass was confirmed to be an ectopic parathyroid adenoma. Successful removal was confirmed by the precipitous drop in serum parathyroid hormone levels postoperatively (Figure 1).

Following successful recovery from the operation, our patient was placed under six-monthly follow-up in the endocrine and clinical genetic clinic, with serial MRI (magnetic resonance imaging) of her abdomen and pelvis. The obstetrics and gynaecology department has been notified of the diagnosis and its strong association with uterine lesions (and infertility), for which she is receiving interval follow-up. An updated pelvic ultrasound has identified three additional intramural fibroids. As HPT-JT syndrome is associated with an increased risk for renal disorders, an MRI (magnetic resonance imaging) of the abdomen was performed, from which multiple renal cysts were identified. Finally, an OPG (orthopantomogram) was performed, which ruled out any jaw lesions.

## 3. Discussion

Inherited primary hyperparathyroidism must be considered (and assessed for) in young patients (below 40 years of age) or in those with a positive family history presenting with primary hyperparathyroidism [3–5]. The estimation of primary hyperparathyroidism is present at one per thousand in the population, and 10% of these cases are considered to be familial; therefore, one per ten thousand cases are due to inherited hyperparathyroidism [2, 4]. Genetic causes of familial hyperparathyroidism include multiple endocrine neoplasia (MEN1, MEN2A, and MEN4), familial isolated hyperparathyroidism, and hyperparathyroidism-jaw tumour (HPT-JT) syndrome [4, 6].

HPT-JT syndrome is inherited in an autosomal dominant fashion [6]. Demonstration of the CDC73 (also known as HRPT-2) gene mutation is diagnostic of the condition [7]; CDC73 is a tumour suppressor gene, involved in regulating the cell cycle (cell division cycle 73) [3]. The CDC73 gene is located on chromosome one and encodes parafibromin, a 531-long amino acid exerting antiproliferative capabilities [3, 8]. Parafibromin promotes cell cycle arrest as well as functioning to regulate the expression of various genes and chromatin structure (via RNA polymerase-II associated factor 1 complex) [9–12].

Due to the scarcity of this condition, a true prevalence cannot be determined; however, estimates include 200 cases reported within the medical literature since its debut in 1958 [13, 14]. Although the disorder is inherited in an autosomal dominant pattern, the penetrance is incomplete (70%); however, it is noted to increase with age [3–5, 15–17]. Furthermore, penetrance is reduced in females for reasons which are unclear [6, 18–20].

The most common manifestation of HPT-JT syndrome is primary hyperparathyroidism, in which single-gland adenoma (typically cystic) involvement is predominant (15% of cases arise from a carcinoma) [3, 6, 12, 15, 19, 21, 22]. A distinction is noted in relation to other familial hyperparathyroidism disorders, which demonstrate multiglandular involvement [3, 23–26]. The mean age at diagnosis of primary hyperparathyroidism in HPT-JT syndrome is 27 (12–58 years) [15, 27], with the earliest recorded case in a seven-year-old patient [15, 28]. The primary hyperparathyroidism may be mild and can often go unnoticed; however, if severe (or the presence of a hypercalcaemic crisis), a carcinoma must be considered [3, 29]. Recurrence of primary hyperparathyroidism is common in HPT-JT syndrome, as demonstrated in our case report [3]. With the prolonged, mild hypercalcaemia, our patient demonstrated, as well as the minimal increase in parathyroid hormone levels and initial normalisation of serum calcium (and parathyroid hormone) postoperatively, these findings are suggestive of benign (and not malignant) primary hyperparathyroidism.

Suggestions for differentiating an adenoma from a carcinoma include parathyroid hormone levels threefold above the upper limit of normal; severe hypercalcaemia; and lesions greater than three centimetres in size [3, 15, 30, 31].

Rather infrequently, children with a CDC73 gene mutation may demonstrate malignant primary hyperparathyroidism [3]. Moreover, a palpable mass will be evident with a carcinoma, in addition to systemic symptoms such as cachexia. As the carcinoma progresses, dysphonia and dysphagia may occur, as well as hypercalcaemic symptomatology (nausea, vomiting, confusion, bone pain, and fractures) [3, 7, 15, 17, 32–34].

Succeeding primary hyperparathyroidism, the next most frequent manifestation involves neoplasms of uterine origin (50%), which can be either benign (leiomyoma, hyperplasia, adenomyosis, adenofibroma, or multiple adenomyomatous polyps) or malignant (adenosarcomas) [3, 4, 16]. Moreover, Mullerian duct tumours have been described [3]. Common to patients with HPT-JT syndrome (and seen in our patient), there is an increased frequency of miscarriages and infertility [3, 35].

Contradictory to the nomenclature, jaw tumours are not prominent in this disorder and are prevalent in no more than 30% of individuals [3]. Better known as “ossifying fibromas,” these lesions (from either the maxilla or mandible) arise from periodontal ligaments within the premolar or molar areas and are typically observed prior to the third decade [3, 15, 19]. Although benign (with a risk of malignant degeneration below one percent) [15, 36, 37], the local destruction of tissue incorporates functional limitations (such as breathing difficulties, dentition abnormalities, periorbital swelling, and cosmetic concerns) necessitating its removal [3, 36, 38]. These lesions can be bilateral and multifocal and may also recur; therefore, monitoring for recurrence is essential [3, 39]. These fibromas can be distinguished from sporadic causes on an X-ray, with HPT-JT syndrome demonstrating radiolucency, as opposed to mixed radiolucency/radiopaque [3, 15, 40].

Less frequent demonstrations include renal anomalies (15–20%) with cysts being the commonest lesions [3, 15]. Other renal anomalies include hamartomatous lesions, Wilms' tumour, mixed epithelium-stromal tumour (MEST), and papillary renal carcinoma [3, 5, 21]. Cysts can vary from minimal lesions to polycystic-like disease presentation (leading to renal failure) [12, 15, 41].

Other uncommon neoplasms have been observed in HPT-JT syndrome; however, debate ensues regarding the increased frequency of some of the neoplasms due to limited available data: pancreatic, testicular, thyroid (Hürthle cell), colon, cholangiocarcinoma, pituitary cysts, and chronic lymphocytic leukaemia [3, 5, 15, 21, 42].

### 3.1. Management

With this infrequent diagnosis, there are no formal guidelines in the diagnosis and management of HPT-JT syndrome; however, genetic analysis is confirmatory [3, 15, 36]. Surgery remains the ultimate management of the primary hyperparathyroidism (total, subtotal, or limited parathyroidectomy); however, there is as of yet no gold standard [3]. Total parathyroidectomy risks hypoparathyroidism (of permanent duration) and is best avoided; limited parathyroid excision is preferred due to an unlikely risk of hypoparathyroidism and decreased morbidity. Total parathyroidectomy has been attempted in the setting of autotransplantation (typically the forearm); however, this can potentially disseminate tumorous cells should a carcinoma be present [3, 31]. If patients are poor surgical candidates, cinacalcet has demonstrated efficacy in treating primary hyperparathyroidism [3, 15]. Should a carcinoma be confirmed, “en bloc” resection is the gold standard, with the removal of the ipsilateral thyroid, parathyroid, fatty tissue, and lymph nodes; a biopsy is contraindicated due to the risk of seeding the tumour [3, 5, 15, 29, 31, 43]. Postoperative complications are best avoided with preoperative treatment of vitamin D (to limit hypocalcaemia) and antiemetics (dehydration promotes devascularisation of the remaining parathyroid gland) [15]; it is imperative to inform the patient, however, that despite the surgical approach used, baseline risk of recurrence (25%) remains for primary hyperparathyroidism [3].

Obstetric and gynaecological manifestations are managed on an individual basis, as are renal anomalies. Female patients are encouraged to attain normocalcaemia prior to pregnancy to prevent foetal and maternal complications (otherwise, likely to require surgery within the second trimester); furthermore, a hysterectomy may be required in the presence of certain uterine lesions; however, this should be a last resort to preserve the potential for conception [3, 15].

Surveillance is recommended from genetic confirmation of diagnosis, including the following [3, 15]:

- Annual calcium, parathyroid hormone, and vitamin D
- Periodic ultrasound examination of the parathyroid gland
- Panoramic X-ray dental imaging (with neck shielding) at least 5-yearly
- Renal imaging (ultrasound/MRI/CT) at least 5-yearly
- Regular gynaecological evaluation (at least pelvic ultrasound)

Family members must be screened for this diagnosis [3, 15, 36]; in the case report presented, our patient was advised to inform her family, who have undergone screening and are awaiting the results. Lifelong biochemical monitoring is mandatory as the risk of recurrence for hyperparathyroidism is around one-quarter, with a second lesion either occurring synchronously or metachronously any time after identification of the first adenoma [3, 5, 15]. Reoperations are considered on an individual basis [44].

This case report further reinforces the limitations of nuclear imaging for the identification of a hyperfunctioning parathyroid adenoma. As with our patient, her parathyroid adenoma was not identified upon the sestamibi-SPECT scan, and a false-positive was noted deep to her thyroid gland (no histopathological abnormality identified). Small adenomas and diminished oxyphil expression are well-documented factors associated with poor localisation with sestamibi scanning [45].

## 4. Conclusion

Although a rare diagnostic entity, hyperparathyroidism-jaw tumour syndrome must be considered in early-onset, familial, atypical, or recurrent primary hyperparathyroidism. Genetic analysis is the gold standard for confirming the diagnosis, and patients must be continually monitored and reassessed to detect recurrence and other systemic manifestations of the disorder. Surgical treatment is the favoured approach to managing primary hyperparathyroidism, and all family members must be screened for the genetic mutation.

With the myriad of phenotypes displayed by hyperparathyroidism-jaw tumour syndrome, patients may initially present to departments other than endocrinology or metabolic medicine, including obstetrics and gynaecology (for infertility), urology (for renal stones), or maxillofacial (for jaw tumours). This case report aims to enhance the awareness of hyperparathyroidism-jaw tumour syndrome and its various associations across all medical domains. Moreover, as with our patient, this report serves as a reminder of the limitations with nuclear imaging (sestamibi) for the detection of adenomas.

## Figures and Tables

**Table 1:** Laboratory results.

Investigations	Level	Reference range	
Corrected calcium (mmol/L)	**2.65**	2.2–2.6 mmol/L	
PTH (pmol/L)	**9.5**	1.3–9.3 pmol/L	
PO_4_ (mmol/L)	**0.7**	0.8–1.5	
Vit D (nmol/L)	99	Optimal: >70 nmol/L	
eGFR (mL/min/1.73 m^2^)	**57**	85–125 mL/min	
Urine Ca-Cr	**0.71, 0.86,** and **0.73**	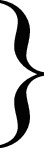	Indicative of increased calcium excretion based on body weight of 50 kg
FE-Ca (%)	**2.58**		
Urine calcium (mmol/24 hour)	**7.16**		

PTH: parathyroid hormone; PO_4_: phosphate; Vit D: 25-hydroxyvitamin D; eGFR: estimated glomerular filtration rate; urine Ca-CR: urinary calcium-to-creatinine ratio; FHH: familial hypocalciuric hypercalcaemia; FE-Ca: fractional excretion of calcium. Bold signifies blood levels outside of the normal reference range.

**Table 2:** Genetic mutations assessed.

Genetic mutation
MEN1
CDC73
CDKN1A
CDKN1B
RET
AP2S1
CASR
GCM2

MEN1: multiple endocrine neoplasia 1; CDC73: cell division cycle 73; CDKN1A: cyclin-dependent kinase inhibitor 1A; CDKN1B: cyclin-dependent kinase inhibitor 1B; RET: rearranged during transfection; AP2S1: adaptor-related protein complex 2 subunit sigma 1; CASR: calcium sensing receptor; GCM2: glial cells missing transcription factor 2.
